# Surface-enhanced Raman scattering spectra revealing the inter-cultivar differences for Chinese ornamental *Flos Chrysanthemum*: a new promising method for plant taxonomy

**DOI:** 10.1186/s13007-017-0242-y

**Published:** 2017-10-30

**Authors:** Heng Zhang, Zhenyi Chen, Taihao Li, Na Chen, Wenjie Xu, Shupeng Liu

**Affiliations:** 1 Key Laboratory of Specialty Fiber Optics and Optical Access Networks, School of Communication and Information Engineering, Shanghai University, 333 Nanchen Road, Shanghai, 200444 People’s Republic of China; 2 Beijing Advanced Innovation Center for Imaging Technology, Capital Normal University, Beijing, 100048 People’s Republic of China

**Keywords:** *Flos Chrysanthemi*, Raman spectra, SERS, Inter-cultivar differences, Plant taxonomy

## Abstract

**Background:**

*Flos Chrysanthemi*, as a part of Chinese culture for a long history, is valuable for not only environmental decoration but also the medicine and food additive. Due to their voluminously various breeds and extensive distributions worldwide, it is burdensome to make recognition and classification among numerous cultivars with conventional methods which still rest on the level of morphologic observation and description. As a fingerprint spectrum for parsing molecular information, surface-enhanced Raman scattering (SERS) could be a suitable candidate technique to characterize and distinguish the inter-cultivar differences at molecular level.

**Results:**

SERS spectra were used to analyze the inter-cultivar differences among 26 cultivars of Chinese ornamental *Flos Chrysanthemum*. The characteristic peaks distribution patterns were abstracted from SERS spectra and varied from cultivars to cultivars. For the bands distributed in the pattern map, the similarities in general showed their commonality while in the finer scales, the deviations and especially the particular bands owned by few cultivars revealed their individualities. Since the Raman peaks could characterize specific chemical components, those diversity of patterns could indicate the inter-cultivar differences at the chemical level in fact.

**Conclusion:**

In this paper, SERS technique is feasible for distinguishing the inter-cultivar differences among *Flos Chrysanthemum*. The Raman spectral library was built with SERS characteristic peak distribution patterns. A new method was proposed for *Flos Chrysanthemum* recognition and taxonomy.

## Background

*Flos Chrysanthemi*, the flower of *Chrysanthemum morifolium* Ramat, was regarded as one of the most much-loved traditional flowers in China with a long history for its surpassingly beautiful look, richness of color, abundant of breeds and wide adaptability. The *Flos Chrysanthemi* recorded in ancient classic of China can be dated back to Spring and Autumn period of Zhou Dynasty in about 3000 years ago. In traditional Chinese and Korean medicines, *Flos Chrysanthemi* has been widely used as herbal medicine in clinical practice for the treatment of colitis, cancer, hypertension, pneumonia, pertussis, and stomatitis [1–7]. Additionally, *Flos Chrysanthemi* was also taken as conventional health food from beverages to spices such as *Flos Chrysanthemi* tea, a popular Chinese drink prepared by dry *Flos Chrysanthemi* in combination with boiling or hot water, and food additive for masking flavors in alcoholic beverages in Korea [8].

As a precious resource gifted by nature for human, *Flos Chrysanthemi* has a very abundant reserve, various type and extensive distribution through wide spread and cultivation around the world. According to investigation statistics, the total amount of *Flos Chrysanthemum* cultivars was estimated as 20,000–30,000 and at least 3000 in China alone [9]. Quite a few studies for *Flos Chrysanthemi* taxonomy in early time depended on relatively conventional methods at the level of morphologic observation and description [10–12]. Conventional taxonomy focused on macroscopic traits in quantitative (plant height, tubular flower diameter, receptacle size, etc.) and qualitative (stem form, petal distribution, receptacle shape, etc.) aspects. Japanese horticulturists usually classify *Flos Chrysanthemum* depend on the practical purpose into edibles and ornamentals two categories, and the ornamentals were divided into small, medium and big three types where the big type were further divided into about 20 subtypes. British gardeners tended to systematize the *Flos Chrysanthemum* into early, medium and late three categories based on flowering time, and in each category they were further divided into 10 subtypes. *Flos Chrysanthemum* classification in the United States was similar to that in Britain, which contained 15 categories.

Several studies that classifying *Flos Chrysanthemum* at chemical or molecular level were reported in recent years. Zhao Y et al. tried to make a classification for 20 varieties of Chrysanthemum by analyzing the peroxidase isozymes (PODs) using polyacrylamide gel electrophoresis (PAGE) [13]. Similarly, another study was reported to have analyzed the PODs in 20 cultivars of *Dendranthema* × *grandiflorum* hybrid groups and found the enzyme spectrums of distinct groups had significant difference, which reflected the genetic diversity of the *Dendranthema* × *grandiflorum* varieties [14]. Besides, a recent research used both PODs and esterase isozyme (EST) analysis to investigate the genetic diversity for 93 cultivars in *Dendranthema* × *grandiflorum* [15]. But the studies were performed with PAGE whose disadvantages of low efficiency and cumbersome operation are obviously: one new technique of high accuracy and efficiency was expected.

Raman scattering, a vibrational spectral technology, was widely applied in the field of material science for substance detection and analysis since it was found by Raman C.V. in 1928 [16]. Theorized as a kind of inelastic light scattering process, Raman scattering has the capacity of characterizing the vibration, rotation, phase transition and crystals structure of molecules specifically so that it could detect overall composition and analyze relative quantification as a fingerprint spectrum. With the development of laser technology and the discovery of enhancement effect by Fleischmann M. in 1974 [17], a breakthrough measurement science termed as surface enhanced Raman scattering (SERS) was born and got a quickly and widely application in material, chemistry and life science field owing to its getting rid of conventional Raman scattering’s shortcoming of rather low intensity. SERS enlarged Raman scattering’s usable scope and was regarded to be an accurate and efficient solution in substance detection and analysis.

In the past few decades, Raman spectroscopic technique was widely used for biological and medical researches, such as bacteria discrimination [18, 19], blood assay [20, 21], and cancer diagnosis [22–24]. While in phytological field, Raman spectroscopic technique was also used to plant identifying and analysis. According to a relevant study, Fourier-transform Raman spectroscopy with 1064 nm excitation was used to discriminate Green Arabica and Robusta coffee beans by monitoring the characteristic Raman bands of kahweol [25]. In another similar research, those two kinds of coffee beans were discriminated using the Fourier-transform Raman spectroscopy by analyzing the extracted lipid fractions [26]. Raman spectroscopy was also reported in discrimination for pollens of different plants. As reported, a set of allergy-relevant pollens, including common ragweed, white birch, English oak, and European linden, were studied using Raman spectroscopy with 633 nm excitation and were successfully distinguished by analyzing and comparing their specific chemical Raman peaks [27]. Raman spectroscopic technique was likely effective in discrimination for different *Flos Chrysanthemum* cultivars. Therefore, in this study, SERS with sliver nanoparticles colloid as enhancement substance was applied to observe the spectral differences among 26 cultivars of Chinese ornamental *Flos Chrysanthemum* and proposes a new method for classifying *Flos Chrysanthemi* by SERS technology.

## Methods

### *Flos Chrysanthemi* samples preparation

All the fresh *Flos Chrysanthemi* corollas, 26 in total, were gifted by the gardening center affiliated with logistics department of Shanghai University. After adequately grinded, the homogenate of each corolla was collected and transferred into a clean centrifuge tube. Then the tubes were centrifuged at 2000 rpm for 10 min and the upper aqueous phases were transferred to fresh tubes as samples for SERS measurement.

### Silver nanoparticles preparation

Sliver colloid was synthesized referring to Lee’s sodium citrate reduction method [28]. Briefly, 1 mL of a 0.1 mol/L sliver nitrate (AgNO_3_, AP, Aladdin) solution was added into 99 mL deionized water (18.3 MΩ*cm, 25 °C) and heated to boiling. 1.9 mL of a 1% sodium citrate tribasic (Na_3_C_6_H_5_O_7_, AP, Aladdin) solution was then added with rapid stirring. The mixture was then kept boiling gently till the color turned celadon. All glassware used were cleaned carefully and thoroughly rinsed with deionized water. The synthesized sliver colloid was checked by Raman scattering before use so as to ensure its availability.

### SERS measurement

Each aqueous phase sample extracted from *Flos Chrysanthemi* corolla was homogeneously mixed with the sliver colloid at the proportion of 1:2 (v/v) and then the mixture was standing for 5 min. A Raman microscope (LabRAM HR Evolution, Horiba, JP) equipped with a 785 nm He–Ne laser device was employed to excite and collect the Raman spectra, in which the initial parameters were configured as follows: exciting power of 3.3 mW (10% of the maximum power of the laser device), resolution of 1 cm^−1^, acquisition time of 10 s and scan range from 400 to 1800 cm^−1^. Every time before measuring, the system was calibrated by 520 cm^−1^ band from silicon reference sample. The laser was focused on the bottom surface of the liquid sample via a L50× microscope objective (N.A. 0.5). Each sample was measured for 6 parallel spectra from different spots to reduce the random error.

### Data processing and analysis

All raw Raman spectra acquisitions were performed with LabSpec software (Horiba, Japan). The baselines of raw data were corrected by Subase V2.10 software (self-developed, HyStudio, Shanghai University, China) with integration of a Vancouver Raman Algorithm based on fifth-order polynomial fitting method [23] for fluorescence background removal.

## Results

**Fig. 1:**
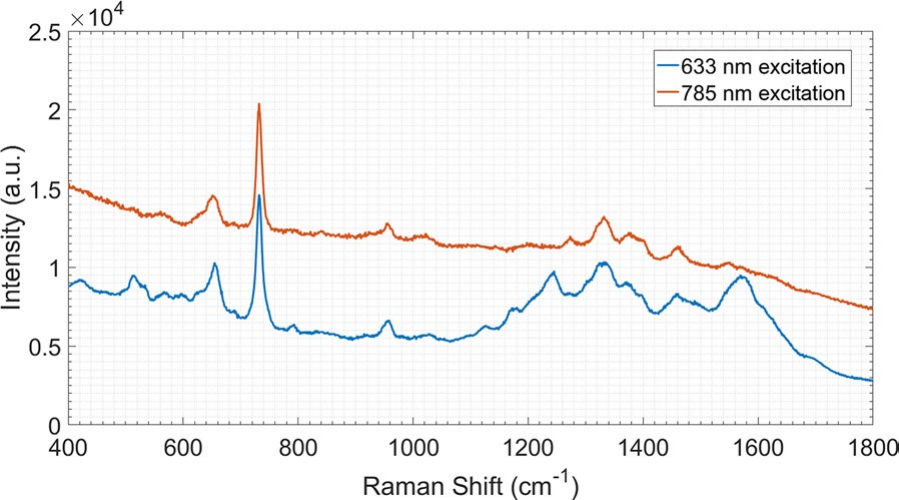
SERS spectra for *Marshal Flag* cultivar with excitation wavelength of 633 nm and 785 nm (10% of maximum laser device power)

The preliminary pre-experiments were performed in order to optimize the experimental conditions and measurement parameters. The frequently-used excitation lasers in conventional Raman measurement for bio-samples were visible light (532 and 633 nm) and near-infrared light (785 nm). In most cases, the shorter the excitation wavelength is, the stronger the fluorescence background will be induced. However, trends are reverse for the thermal effects: longer-wavelength laser might heat the samples more strongly, especially the bio-samples. Therefore, although the shorter-wavelength lasers, like 532 and 633 nm light, were relatively poor in heating bio-samples, the fluorescence background they induced might be a non-negligible interference for the Raman spectral measurement. While, the longer-wavelength lasers, such as 785 nm light, were efficient with lower fluorescence background, but the robust thermal action on bio-samples might potentially limited their usage (irradiation power and irradiating time). For these reasons, an appropriate excitation wavelength and power were rather vital for achieving better measurements. We tentatively conducted the Raman spectral measurement for *Marshal Flag* cultivar using the 633 and 785 nm lasers to test and compare the fluorescence interferences. The result showed that the fluorescence background induced by 633 nm excitation light was stronger than that of 785 nm laser: the baseline was raised in the range from 1100 to 1700 cm^−1^ (Fig. 1). Since higher fluorescence background will bring about more difficulties for baseline correction and affect the reliability of spectral data, 785 nm light was chosen as the excitation laser for the Raman spectral measurement.

**Fig. 2:**
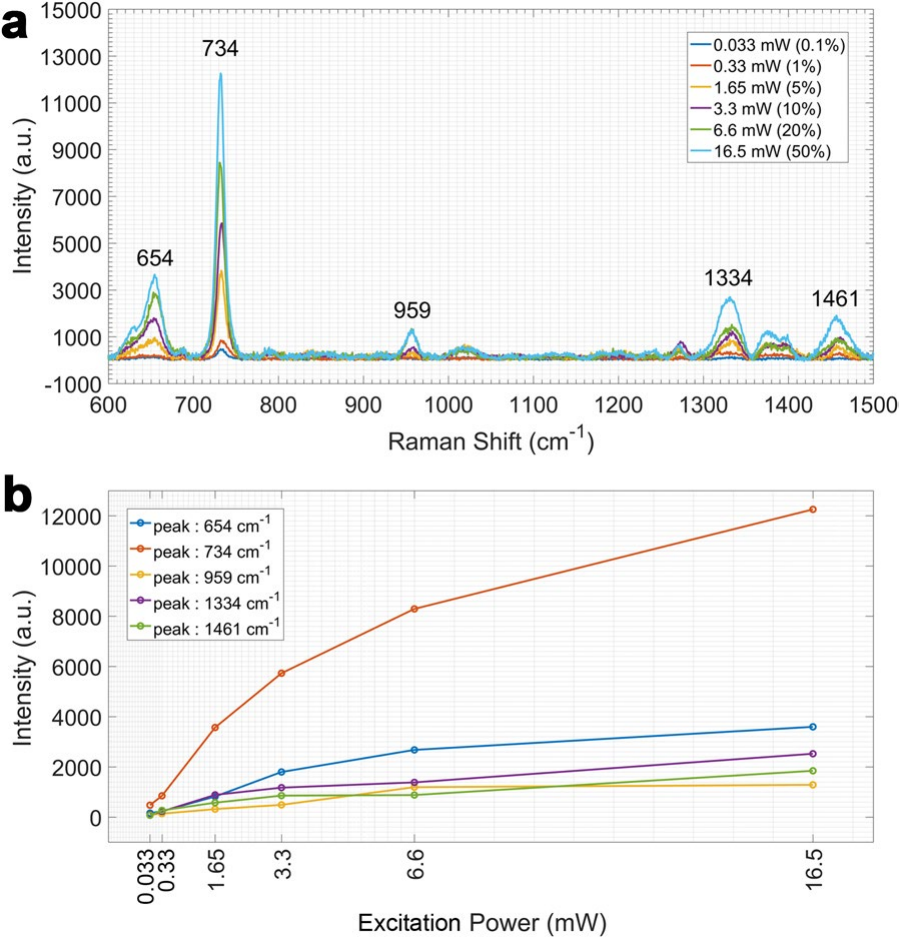
The tentative relationship between the laser excitation power and SERS spectra. **a** The SERS spectral intensity goes with the laser excitation power (for instance of the *Blue Eye* cultivar, 785 nm excitation). **b** The growing trends of intensities for individual peaks might be related to the laser excitation power (for instance of the *Blue Eye* cultivar, 785 nm excitation)

To minimize the thermal damage to the samples, the 785 nm laser should be used in a proper power: higher power would destroy the active components in the samples while much lower power can’t acquire adequate spectral intensities. 0.033, 0.33, 1.65, 3.3, 6.6 and 16.5 mW excitation lasers of 785 nm (corresponding to the 0.1%, 1%, 5%, 10%, 20% and 50% of the maximum laser device output power, respectively) were used in Raman spectral measurement for *Blue Eye* cultivar as an example. The result showed that the overall Raman spectral intensities seemed to grow with the increase of laser power (Fig. 2a). In further analysis for individual peaks, this growth trends were not stable in all power level. In lower power levels (0.033 and 0.33 mW), the intensities were rather weak so that the random noise signals made them difficult to represent real spectra. And in higher power levels (6.6 and 16.5 mW), the intensity in peak 1461 cm^−1^ was slightly decrease at 6.6 mW power level, compared with the 3.3 mW level; the intensity in peak 959 cm^−1^ was slightly stronger at 6.6 mW power level than that at 16.5 mW power level (Fig. 2b). Moreover, the intensity growth trends of 959 and 1461 cm^−1^ at 6.6 and 16.5 mW power levels were not consistent with that at the middle power levels (1.65 and 3.3 mW). Those typical abnormal relationships between Raman spectra and the excitation power in higher levels might be attributed to that the thermal effect of higher power laser caused some certain impacts on some certain components in the bio-samples. In addition, the intensities at 3.3 mW power level were higher than that at 1.65 mW and Raman spectra were adequate for measurement. Therefore, in comprehensive consideration, the 3.3 mW 785 nm laser was used as the excitation light for the Raman spectral measurement.

With the enhancement of sliver colloid, all SERS spectra of 26 cultivars were measured and their fluorescence backgrounds were removed by baseline subtraction. For each sample, the post-correction spectra signal data were averaged to be the mean spectrum and the characteristic peaks were extracted by picking out the distinct and tangible protuberances in spectral curves (height more than 400 a.u. and width less than 100 cm^−1^). All characteristic peaks of every sample were normalized by the max value among them so that the distribution patterns, including the relative heights (vertical axis) and positions (horizontal axis) in the measured range could be abstracted and recorded into a data library as the references for distinguishing different cultivars (Table 1). In order to make the characteristic peaks distribution patterns observed visually, the normalized peaks data of all the samples were mapped into a color block image, where the value range from 0 to 1 corresponded to the hue from violet to red (Fig. 4).

**Table 1:** Raman spectral characteristic peaks and normalized intensities of all 26 *Flos Chrysanthemum* samples

Common cultivar name	Characteristic peaks (Raman shift, cm^−1^) and normalized intensities											
*Golden Queen*	Peaks	569	624	652	731	958	1324	1462				
	Normalized intensities	0.049	0.046	0.155	1	0.136	0.171	0.078				
*Madame Guo*	Peaks	651	731	958	1018	1333	1460					
	Normalized intensities	0.175	1	0.101	0.084	0.221	0.099					
*Flying Ziyan*	Peaks	657	733	1026	1275	1335	1459					
	Normalized intensities	0.208	1	0.084	0.095	0.220	0.135					
*Fenghuang*	Peaks	647	731	1334								
	Normalized intensities	0.150	1	0.198								
*Red Frost*	Peaks	654	733	954	1023	1272	1334	1459				
	Normalized intensities	0.266	1	0.093	0.093	0.095	0.199	0.135				
*Red Jinbei*	Peaks	654	731	957	1017	1334	1461					
	Normalized intensities	0.158	1	0.131	0.072	0.192	0.084					
*Marshal Flag*	Peaks	567	654	732	959	1273	1333	1377	1461	1550		
	Normalized intensities	0.057	0.263	1	0.098	0.069	0.209	0.104	0.120	0.056		
*Jade Feng*	Peaks	516	568	656	732	957	1174	1201	1331	1377	1455	1547
	Normalized intensities	0.069	0.084	0.333	1	0.102	0.067	0.082	0.280	0.071	0.186	0.083
*White Jade*	Peaks	512	564	654	732	958	1022	1329	1377	1457		
	Normalized intensities	0.065	0.065	0.266	1	0.114	0.0422	0.203	0.061	0.112		
*White Lion*	Peaks	564	657	732	957	1020	1271	1333	1373	1459		
	Normalized intensities	0.064	0.182	1	0.078	0.060	0.089	0.227	0.082	0.138		
*Golden Lotus*	Peaks	656	733	957	1333	1374	1461					
	Normalized intensities	0.275	1	0.097	0.220	0.083	0.142					
*Green Zhaoyun*	Peaks	570	654	732	959	1022	1270	1333	1376	1399	1457	
	Normalized intensities	0.051	0.273	1	0.115	0.055	0.049	0.203	0.104	0.087	0.138	
*Gushui Liuxia*	Peaks	563	657	733	957	1021	1272	1333	1379	1401	1457	
	Normalized intensities	0.065	0.287	1	0.102	0.084	0.081	0.209	0.106	0.082	0.155	
*Green Window*	Peaks	515	567	657	732	958	1173	1245	1334	1457	1578	
	Normalized intensities	0.133	0.062	0.351	1	0.134	0.064	0.180	0.148	0.142	0.342	
*Fireworks*	Peaks	649	731	958	1331	1403						
	Normalized intensities	0.112	1	0.136	0.218	0.069						
*Golden Needle*	Peaks	733	1331	1375	1459	1576						
	Normalized intensities	1	0.216	0.104	0.107	0.145						
*Blue Eye*	Peaks	654	734	959	1273	1334	1461	1549				
	Normalized intensities	0.288	1	0.080	0.126	0.198	0.157	0.076				
*Clean Water Hehua*	Peaks	567	653	732	957	1274	1333	1372	1462			
	Normalized intensities	0.065	0.249	1	0.118	0.064	0.202	0.059	0.115			
*Mini Ju*	Peaks	655	733	957	1018	1278	1331	1463				
	Normalized intensities	0.260	1	0.113	0.098	0.086	0.211	0.156				
*Gold Ball*	Peaks	513	595	656	733	1223	1333	1377	1459	1576		
	Normalized intensities	0.115	0.077	0.183	1	0.088	0.227	0.076	0.119	0.211		
*Beautiful Purple*	Peaks	732										
	Normalized intensities	1										
*Beijing Red*	Peaks	733										
	Normalized intensities	1										
*Mini Red Peach*	Peaks	731	1335									
	Normalized intensities	1	0.188									
*Afric Ju*	Peaks	649	731	957	1335							
	Normalized intensities	0.230	1	0.130	0.212							
*White Powder*	Peaks	568	651	732	896	957	1272	1334	1372	1457		
	Normalized intensities	0.073	0.188	1	0.078	0.092	0.065	0.213	0.077	0.117		
*Golden Mudan*	Peaks	651	732	959	1326	1383	1399	1459				
	Normalized intensities	0.187	1	0.132	0.223	0.081	0.082	0.115				

**Fig. 3:**
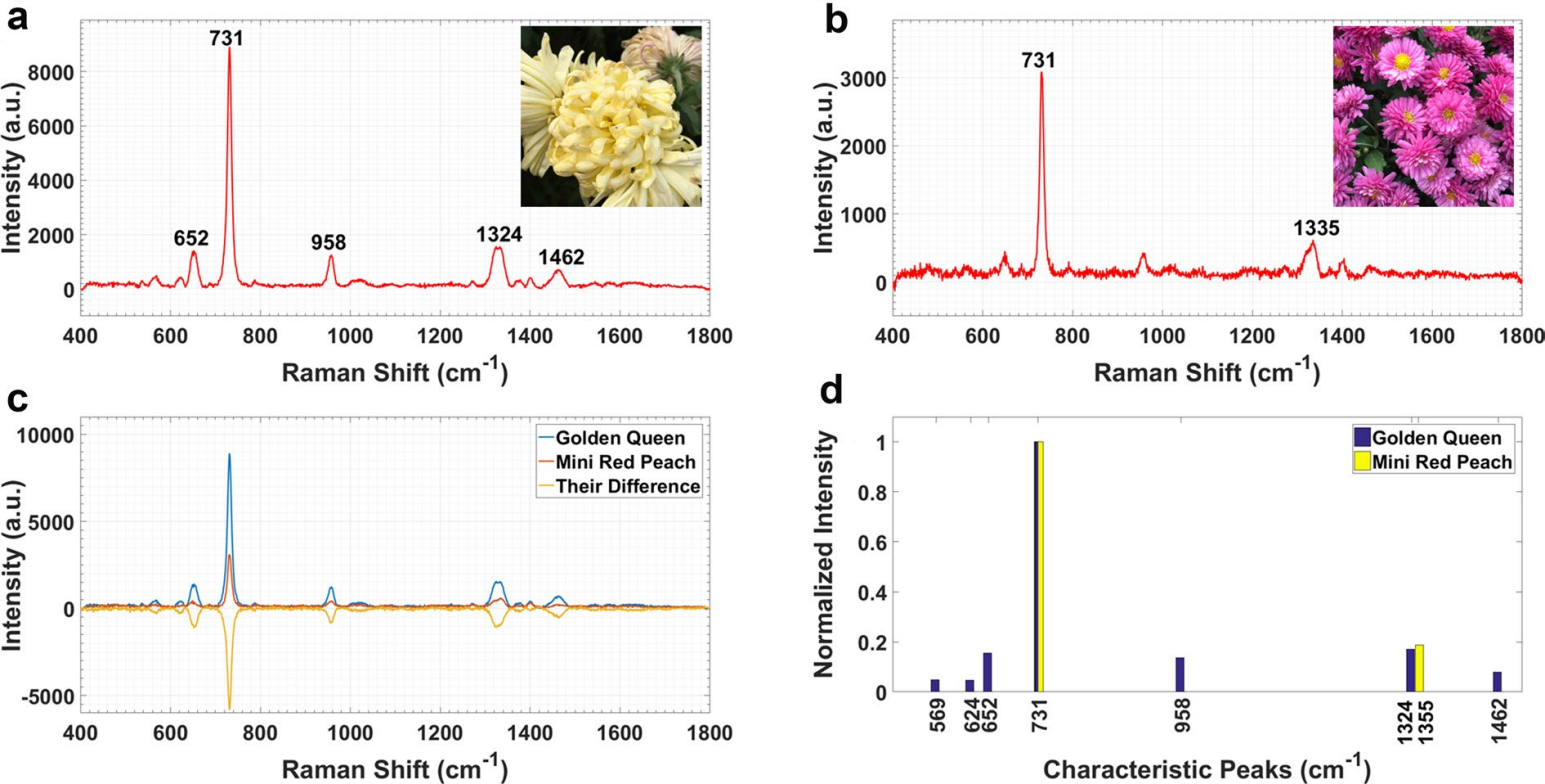
SERS spectra reveal the differences among cultivars of *Flos Chrysanthemum*. **a** Spectrum and image of *Golden Queen* cultivar. **b** Spectrum and image of *Mini Red Peach* cultivar. **c** The differences between these two spectra. **d** The contrast between their distribution patterns of normalized characteristic peaks

**Fig. 4:**
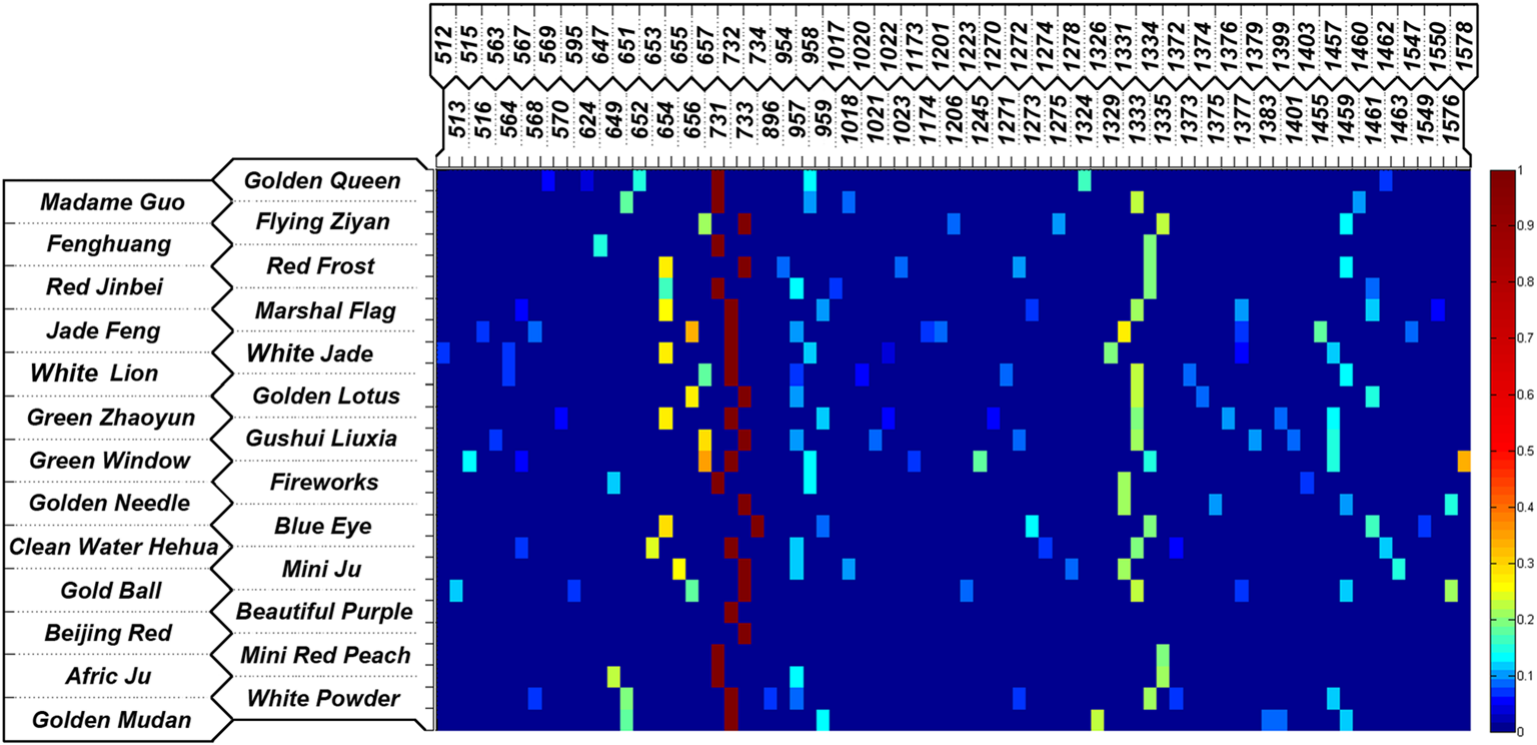
Characteristic peaks distribution patterns map for all 26 *Flos Chrysanthemum* samples

*Golden Queen* and *Mini Red Peach* breed are taken as examples to illustrate how to parse the inter-cultivar differences by SERS spectra. In spite of the similarity lying in the trend and shape between these two spectra (Fig. 3a, b), their detailed traits including peak heights make obvious differences (Fig. 3c). In addition, their characteristic peak distribution patterns could contribute to distinguishing and matching the different cultivars via less dimensionality (Figs. 3d, 4).

**Fig. 5:**
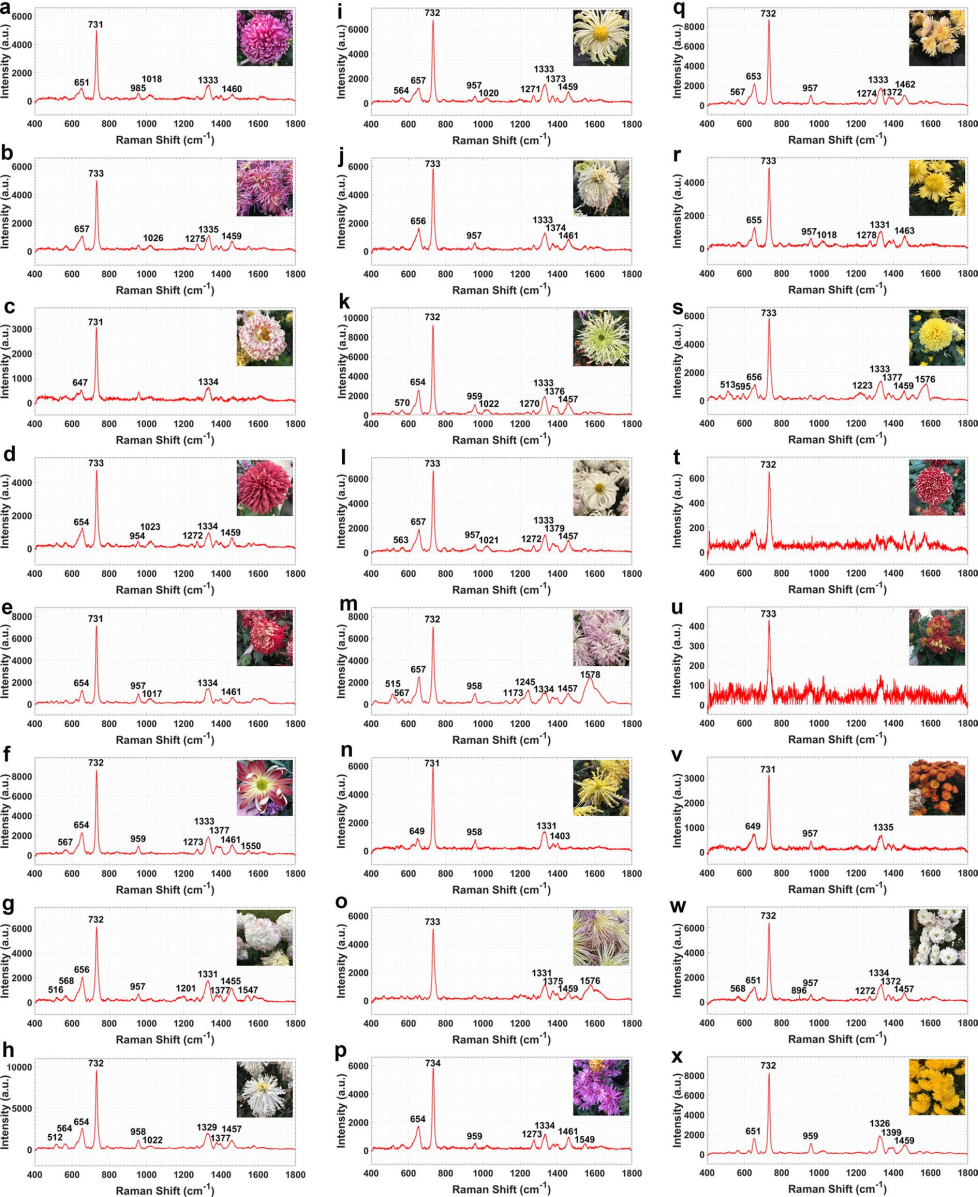
Part of SERS spectra and images from the spectral library of Chinese ornamental *Flos Chrysanthemum*. **a** *Madame Guo*. **b** *Flying Ziyan*. **c** *Fenghuang*. **d** *Red Frost*. **e** *Red Jinbei*. **f** *Marshal Flag*. **g** *Jade Feng*. **h** *White Jade*. **i** *White Lion*. **j** *Golden Lotus*. **k** *Green Zhaoyun*. **l** *Gushui Liuxia.* **m** *Green Window.* **n** *Fireworks*. **o** *Golden Needle*. **p** *Blue Eye*. **q** *Clean Water Huhua*. **r** *Mini Ju*. **s** *Gold Ball*. **t** *Beautiful Purple.* **u** *Beijing Red*. **v** *Afric Ju*. **w** *White Powder*. **x** *Golden Mudan*

Except for these two cultivars, the rest in the spectral library are shown in Figs. 4, 5 and Table 1. It can be viewed in the characteristic peaks distribution patterns map (Fig. 4) and spectral library (Fig. 5) that the general outlines and trends of spectra from all samples were similar in a broad scale; after all, they belonged to the same species and contained semblable chemical constituents. Almost all spectra contained a remarkable peak with the strongest scattering intensity in the range of 731–734 cm^−1^, which might be assigned to the gallates [29], a kind of antioxidant phenolic esters contained in *Chrysanthemum* [30]. In addition to the 731–734 cm^−1^ bands, the characteristic peaks distribution patterns map (Fig. 4) showed another 4 obvious bands of peaks in the vertical angle of view (651–657, 954–959, 1324–1135 and 1455–1462 cm^−1^). The characteristic peaks in range of 651–657 cm^−1^ could be assigned to the 1,8-Cineol (δ-ring vibration), a kind of disaccharides in *Chrysanthemum* [31] and the weaker bands in 954–959 cm^−1^ might be assigned to the carotenoid [32]. Bands from 1324 to 1135 cm^−1^ might be attributed to the chlorophyll a and b, which were found in most parts of the plants [33, 34]. The peaks distributed in range of 1455–1463 might be assigned to the sucrose, which widely existed in plants [35, 36].

Apart from the comparatively universal peaks in most cultivars described above, there also existed other peaks which were unique for specific cultivars and that might be just the reason why they differed. The cultivar *White Lion* had a characteristic peak (1020 cm^−1^) in it, which could be assigned to the crocetin, making itself distinct from others. If take measurement errors in consideration, *Red Jinbei* (1017 cm^−1^), *Madam Guo* (1018 cm^−1^), *Mini Ju* (1018 cm^−1^), *Gushui Liuxia* (1021 cm^−1^), *Green Zhaoyun* (1022 cm^−1^), *White Jade* (1022 cm^−1^) and *Red Frost* (1023 cm^−1^) could also be tentatively inferred to contain crocetin because the peaks were close to 1020 cm^−1^ in an acceptable range. The characteristic peaks distribution patterns abstracted from the SERS spectra varied from cultivars to cultivars. For the bands distributed in the pattern map (Fig. 4), the similarities in general showed their commonality while in the finer scales, the deviations and especially the particular bands owned by few cultivars revealed their individualities. Since the Raman peaks could characterize specific chemical components, those diversity of patterns could indicate the inter-cultivar differences at the chemical level in fact. Furthermore, a SERS spectral library, containing the images, spectra and the corresponding characteristic peak distribution patterns of all 26 cultivars of Chinese ornamental *Flos Chrysanthemum* within this study, was built in order to provide convenience for *Flos Chrysanthemum* finding, referring, recognition and taxonomy researches.

## Discussion

Raman scattering, a vibrational molecular spectroscopic technique, has emerged as one of the major tools in biological application, especially the components detection and identification. In Raman spectra analysis, the Raman shift is unique to individual molecules topically while globally the Raman spectrum, the plot of scattering intensity as a function of wavenumber, varies for different components. In finer spatial scales, the peaks in spectrum could always correspond to particular chemical bonds in the molecule, for which the Raman spectrum was regarded as a molecular specific fingerprint spectrum to provide direct information from chemical components. Compared with other conventional methods used for substance and material analysis, Raman scattering technique was relatively simple, reproducible, nondestructive and only a small part of samples were needed. In view of these advantages, we tried to use this technique to analyze the inter-cultivar differences in *Flos Chrysanthemum*.

The surface-enhanced Raman scattering (SERS), an enhancing effect for Raman scattering by rough metal nanoparticles [17], could overcome the shortcoming of low sensitivity in conventional Raman spectroscopy and became a rather mature technology for substance detection and analysis. In this study, we used sliver colloids as enhancement substrate for SERS measurement and achieved relative good enhancement effects.

Since a clear and narrow spectral peak corresponds to a specific vibrational bond in molecules, in many cases a combination of several peaks could represent a certain molecule. However, probably not all the bonds in a molecule would be sensitive to Raman scattering, even only one spectral peak being observed mostly. In general, bio-samples such as cells, tissues and secretions, contained numerous kinds of molecular components liked proteins, nucleic acids, lipids, amino acids and etc.. Wherein the macromolecules were synthesized by a combination of small molecules; for instance, the proteins were made of a variety of amino acids. And moreover, many cases existed that the same bond distributed in different molecules; for example the π-bond vibration of aromatic ring laid in both tryptophan and tyrosine. Therefore, it was essential to select a unique bond vibration as the characteristic peak to make the similar molecules distinct.

Thanks to the set of tentative peaks assigned for part of bio-components in plants provided by the published reports (Table 2), it is convenient for us to infer the underlying specific molecules according to the peaks in Raman spectrum. It should be noted that the peak was in a narrow range rather than an exact value because the system errors and random errors could not be avoided. The system errors resulted from instrumental effects might lead to the difference between the value measured in this study and in the referenced literature. The difference of instruments and the calibration methods might impact on the Raman shifts of the same material [37]. And the random errors caused by sample effects might make the value fluctuant in a range. For instance, the condition temperature could influence the Raman shifts to some extent [38]. Besides, the Raman spectrum was a comprehensive and superposed plot for all scattering lights from a mixture of all bio-matters participating in the interaction. Scattering spectrum of each active vibrational bond or molecule superposed other spectra, making the resultant spectral peaks offset from the original position. Therefore the peak gap between the same bonds in the mixed bio-matters and the pure substances sounded reasonable.

**Table 2:** Tentative Raman spectral peak assignments for metabolites in plants [50]

Compounds	Representatives	Peaks (cm^−1^)	Assignment
Amino acids	Cysteine	512	***ν***(S–S) ***g*** **–** ***g*** **–** ***g***
	Methionine	630–670	***ν***(C–S) ***g***
		700–745	***ν***(C–S) ***t***
Proteins	Disordered structure (solvated)	1245	
Lipids/fatty acids		800–1100	***ν***(C–C)
Disaccharides	Sucrose	1462	***δ***(CH_2_)
Bicyclic monoterpenes	1,8-Cineol	652	***δ***(ring)
	Sabinene	652	***δ***(ring)
Tetraterpenes	Crocetin	1020	***ρ***(C–C)
Chlorophyll	Chlorophyll a, b	1326	
	Chlorophyll a	1549	
Carotenoid		956–957 [32]	

Vibrations: *ν*—stretching; *δ*—deformation; *ρ*—in-plane rocking. Conformations: *g*—*gauche*; *t*—*trans*

The inter-cultivar differences among *Flos Chrysanthemum* naturally resulted from the differences of matters they contained, and these differences involved in both substances types and their proportions. Ingredients and their proportions in *Flos Chrysanthemum* varied from cultivars to cultivars, leading to offsets of peaks in spectra. This might be the part reason why the peak of same vibrational bond fluctuated within a narrow range instead of keeping the same value, which made the characteristic peaks distribution patterns in the map looked irregular (Fig. 4). Although the ingredients and chemical components of the 26 cultivars were similar as the same species, their relative proportions differed and the differences were embodied in the distinctions of characteristic peaks distribution patterns. Along with the specificity and uniqueness for several peaks in individual cultivars, the diversity of patterns jointly resulted in the inter-cultivar differences.

Currently, vibration spectral techniques, mainly the infrared spectroscopy and Raman spectroscopy, were being widely applied in plant-related identification, analysis and characterization. In a recent study, the infrared spectroscopy was used as an inexpensive and rapid method to characterization plants by their pollens. The spectroscopic-based methodology was able to detect the phylogenetic variations, including the separation of confamiliar and congeneric species [39]. Another similar research was reported to characterize the aeroallergens by measuring the spore and pollen samples using single reflectance attenuated total reflectance Fourier transform infrared spectroscopy (SR-ATR FTIR) [40]. Also as a kind of vibrational spectroscopic technique, Raman spectra could provide complementary information to that obtained by infrared spectra.

The fingerprint Raman spectrum has capacity of charactering the specific vibration, rotation, phase transition and crystals structure information for individual molecules by their unique vibrational spectra. Based on such features, Raman spectroscopic analyses in principle allow to discriminate different species, and even to make classifications among the same species. In the past few decades, numerous studies have been reported on the application of Raman spectroscopy in botanical researches, including the content measurement for fatty oils [41–43], proteins [44], carbohydrates [45], phenolic [46], alkaloids [47], essential oils [48], carotenoids [49], polyacetylenes [50] and etc.. According to a relevant research, the combinational method of Fourier-transform Raman and Fourier-transform infrared was used to study the plant cell walls and achieved comprehensive spectroscopic information [51]. A very recent study reported that 34 types of pollens underwent Raman spectral measurement and a pollen spectra primary library was built for detecting and identifying pollen in airborne samples, providing an innovative idea and a promising line of investigation for future Raman technology development in the area of aerobiology [52]. As a matter of fact, the method and thought of our study were similar to theirs: we are aiming to build a primary Raman spectral library for corollas of *Flos Chrysanthemum*. Therefore, in principle, a library including image, Raman spectrum and characteristic peaks distribution pattern of each recorded cultivar could be built to provide potential assistances for recognition and taxonomy related researches. And every new cultivar to be found in the future could be documented in the library by archiving its image, Raman spectrum and characteristic peaks distribution pattern according to the method above. It might have very potential and promising application prospects for plant taxonomy related researches to build, supplement and improve the library in the long run. In addition to the vibration spectral techniques, the hyperspectral imaging was reported as a promising technique for plants identification. The hyperspectral imaging technique was used for nondestructive and fast determination of pigment composition and contents of chrysanthemum. The study revealed the differences inter 160 varieties of chrysanthemum by the hyperspectral measurement of the color phenotypic and pigment contents [53]. The inter-cultivar differences among *Flos Chrysanthemum* were analyzed in this study by the comprehensive Raman spectra for all Raman-active matters in corollas, which might be also another promising method for plant taxonomy.

The results showed that SERS spectroscopy was potentially capable in the discrimination and identification of the *Flos Chrysanthemum* by corollas at the cultivar level. The differences in the characteristic peaks distribution patterns at finer scale suggested that there were enough variances among corolla biomolecules that allowed cultivars separation. Plant corollas contained various chemical components, such as chromatoplasm, aromatics, amino acids and polysaccharides. Each of them was distinct in molecular conformations and they interacted with the adjacent molecules in very different ways. The enormous complexity made it nearly impossible to completely assign every spectral band to the individual component. Nevertheless, if it was unnecessary to clearly learn the details of each component they contained, this complexity could be utilized in turn to characterize and discriminate the plants. The plant taxonomy was a much more comprehensive multi-discipline involving morphological, cytological, ecological and genetic characteristics of plants [51]. It was generally recognized that the more characteristics the classification used, the more predictable it would be. Since the Raman spectra could represent not only the type and amount information of the molecules but also the information of molecular conformations and the interacting relationships with the adjacent molecules, it might be a promising method for plant taxonomy.

Potential limitations for this methodology in the study were expected to be analyzed and discussed for further improvement. SERS spectra from other parts and spots of the *Flos Chrysanthemum* for each cultivar needed to be acquired as supplement information to improve the comprehensiveness of library. A more comprehensive and detail archive could be of benefit to the accuracy for cultivar identification and expanding our knowledge. In addition, more cultivars remained to be measured and documented to enlarge the library. However, this was a pilot study to try charactering the inter-cultivar differences and making classifications for 26 cultivars of Chinese ornamental *Flos Chrysanthemum* within our reach, and this new methodology might be promising for inducing more valuable findings and innovations in future.

## Conclusion

SERS technique is feasible for distinguishing the inter-cultivar differences among *Flos Chrysanthemum*. A Raman spectral library with images, spectra and characteristic peak distribution patterns was built, and this work presented a new promising method for *Flos Chrysanthemum* recognition and taxonomy.

## Abbreviations

SERS: surface-enhanced Raman scattering
PAGE: polyacrylamide gel electrophoresis
POD: peroxidase
EST: esterase
AP: analytically pure
a.u.: arbitrary unit
ect.: et cetera
